# Lectures on Inflammation and Ulceration of the Cervix Uteri—In Answer to the Croonian Lectures of Dr. Charles West, on the Same Subject

**Published:** 1855-01

**Authors:** Henry Miller

**Affiliations:** Professor of Obstetric Medicine in the Medical Department of the University of Louisville


MEDICINE AND SURGERY.

JANUARY, 1855. NEW SERIES. VOL. III.—No. 1.

### ART. I.—LECTURES ON INFLAMMATION AND ULCERATION OF THE CERVIX UTERI—IN ANSWER TO THE CROONIAN LECTURES OF DR. CHARLES WEST, ON THE SAME SUBJECT.

BY HENRY MILLER, M. D., PROFESSOR OF OBSTETRIC MEDICINE IN THE MEDICAL DEPARTMENT OF THE UNIVERSITY OF LOUISVILLE.

### LECTURE I.

THE Croonian Lectures for the year 1854 were assigned to Dr. CHARLES WEST, of London, who selected as his theme "An Inquiry into the Pathological Importance of Ulceration of the Os Uteri,"—and to the discussion of the subject he devoted three lectures, which make in print an octavo volume of ninety-five pages. These lectures have been favorably received by the profession in England, and have just been republished iu this country in the *Medical News and Library*, heralded by the *American Journal of the Medical Sciences.*

The liability of the os uteri to such a lesion was either unknown or but slightly regarded until the *speculum uteri* gave ocular proof of its reality, and suggested the application of topical remedies for its cure. Then as always before, when a novel method of investigation is introduced, or a new mode of treatment proposed, it was not to be expected that the innovation would be adopted, until it had received the sanction of experience and the stamp of criticism. Accordingly the speculum and its revelations, its uses and abuses, have given rise to much animated discussion and, in not a few instances, to acrimonious criticism, mingled with personal abuse and downright blackguardism.

In the temper and phraseology of these lectures of Dr. WEST, I find nothing that is exceptionable. His style is chaste and his manner is courteous and dignified. But the means which he has resorted to to disparage and, to the extent of his abilities, render contemptible the doctrine and practice of those from whom he differs, are not so laudable. After the most careful perusal of his lectures, my deliberate judgment is, that for whatever force or point they may possess, they are indebted to such a partial statement of the doctrine oppugned as amounts to actual misrepresentation. Not that Dr. WEST is ignorant of the doctrine he has undertaken to explode, or that he has failed to set it forth, once and again, in various parts of his lectures. But these full and explicit statements of it appear only in the course of his discursive remarks and in an incidental manner. When they are made to assume the distinct and substantive form of a proposition, which is to be sustained or refuted, then the resort to the artifice of a mutilated statement may be easily discovered, and to this and this alone he owes the apparent victory which he achieves in the controversy.

Nor are these lectures free from the vice of dissimulation. When Dr. WEST wings an argument and succeeds, to his satisfaction, in infixing its point in the obnoxious doctrine, he is wont to assume a philosophical air, and caution his hearers against yielding too implicitly to its force, for after all, the doctrine which it seems to refute may be valid and its validity may perhaps be established by counter evidence of greater potency. This apparent ingenuousness and, as I must regard it, affectation of philosophical caution and distrust, mightily predisposes the reader to submit himself to the guidance of Dr. WEST and quietly to adopt whatever conclusions he may reach.

To justify these general reflections, let us proceed to a critical examination of the work before us.

The first lecture of Dr. West opens with some pertinent allusions to the more usual sources of the sexual disorders of females, which he finds in the situation, structure and functions of the womb. By its situation at the lowest part of the trunk and the absence of valves from its veins, the return of blood from the organ is rendered difficult, while it is subject, every month, to sanguinous inundations which are abated by the increased natural secretions of its internal surface and the hemorrhage which "breaks forth along the whole tract." The hemorrhage may be protracted or checked by slight causes, and, in either case, derangement of the organ and impaired health of the individual may be the consequence.

Again: in the laxity of the ligaments that support the uterus and their liability to be weakened by the causes, which increase the weight of the body they have to bear, is found a cause of displacement of the organ, especially of prolapsus. And when prolapsed, it is exposed to disturbing and irritating influences, from which it was exempt while suspended in the pelvic cavity.

The last source of disorders of the womb, pointed out by Dr. West, is furnished by the changes that attend upon conception and parturition. I need not follow him in his brief but accurate description of these changes. It is sufficient to say, that he finds, and justly I think, in all causes that interrupt the return of the uterus, after delivery, to its unimpregnated condition, a most fruitful source of disease. It is apt to remain permanently increased in size, the menstrual function is ill-performed, its secretions differ in quantity and quality from those of its healthy state, all the sexual functions are apt to be performed with pain, impregnation is less likely to occur, and if pregnrncy should take place, there is great probability of its coming to a premature termination.

"This set of symptoms, however," as Dr. West very properly remarks, "or at least many of them, are met with independent of pregnancy and its consequences, supervening sometimes, indeed, under the influence of causes which evidently, and in a marked manner, interfere with the generative functions, but coming on at other times slowly, and, as far as we can discover, without cause. How are they to be explained? Do they proceed from an invariable pathological occurrence, which is present in every case, how wide soever may be in other respects the points of difference between them—or are they the indications of disordered function, which may depend on causes as various as those which produce vomiting or occasion dyspnœa? The enquiry is manifestly an important one; its elucidation will be the object of these Lectures. It has been said that there is an invariable, or almost invariable, cause of these symptoms,—that, be the remote occasion of them what it may, inflammation and ulceration of the neck of the womb is their immediate cause,—that the key to the right understanding of uterine diseases is to be found in the correct appreciation of the importance of this condition; and the cardinal point in their treatment consists in the adoption of means for its cure."

Dr. WEST next describes the "ulcerations" to which such important results are attributed, and I shall offer no particular objection to his description. They are, in truth, for the most part, "mere superficial abrasions of the epithelium investing the lips of the os-uteri," and they do "seldom or never present an excavated appearance with raised edges, as ulcers of other parts often do." But when he speaks of these ulceraticns as of such weighty moment, in the estimation of some, he does injustice to the opinions of those with whom he differs, for I will venture to affirm that no writer on this subject can be referred to, who attaches any pathological value to these superficial abrasions, apart from the inflammation of which they are merely the effects. They are the product, but not the invariable sequence of infiammation. Inflammation may exist, and that too in great intensity, without ulceration, and whether ulceration attend or not is of trivial importance, as it demands little or no special treatment and does not materially add to the gravity of the symptoms. The essential disease is *inflammation*, and it is unfortunate, I think, that the accompanying lesion of structure, which may or may not attend, has attracted notice beyond its deserts, seeing that it is calculated needlessly to alarm patients thus afflicted, and has been the occasion of unprofitable verbal disputes.

In the quotatiou which has just been made, Dr. WEST, it will be observed, does full justice to the doctrine he is combating by coupling inflammation with ulceration; but in the immediately succeeding paragraph, descriptive of the ulceration, he is guilty of the unfairness I have charged against him, in alleging that it is ulceration only which is the *summum malum* of those whom he is opposing; and here commences his mighty effort, which is continued with unabated force throughout his lectures, to depreciate and ridicule these ulcerations, by such expressions as the following:—"seemingly trivial ulceration of the os uteri," "some slight abrasion of the mucous membrane covering this part," "trifling abrasion or ulceration of the os uteri," as if those whose views he is controverting verily believed that a lesion, of no greater consequence than a pin scratch, can mar the functions of the entire sexual apparatus and project its malign influence to distant organs in sympathy with it.

It is this resort to an equivoke—using the term "ulceration" as an equivalent for inflammation and ulceration, but expecting the reader to understand by it nothing more than an insignificant abrasion, which gives to Dr. West an unfair advantage in the argument—a logical trick, which he practices throughout his lectures and is well calculated to bewilder his reader.

Having described the morbid appearances of the os uteri, when brought under ocular examination by the speculum, and referred to their paramount importance in uterine pathology, in the estimation of some, Dr. West proceeds to propound the question which he has undertaken to discuss, viz:—"Whether ulceration of the os uteri is to be regarded as the first in a train of processes which are the direct or indirect occasion of by far the greater number of the ailments of the generative system; or whether, on the other hand, it is to be considered as a condition of slight pathological importance, and of small semeiological value,—a casual concomitant, perhaps, of many disorders of the womb, but of itself giving rise to few symptoms, and rarely calling for special treatment?"

Here I beg you to observe that the trick—the equivoke—which has been exposed, is had recourse to in the very statement of the question to be discussed. If by "ulceration" of the os uteri Dr. West means a trifling abrasion, independent of precursory and accompanying inflammation, all argumentation is precluded, for it would be a waste of time to combat such a spectre—such a mere pathological ghost. Inflammation, I repeat it, is the essential disease, and ulceration is but an accident of no pathological or therapeutical value.

Dr. WEST next proceeds to adduce the allegations by which the former opinion, namely, that which rates highly the importance of ulceration of the os uteri, is supported; and then to remove all suspicion that the whole of what can be urged in its favor may not have been frankly and fully brought forward, he makes the following affirmation:—"This picture (and I have added nothing to its coloring) of all the ills which follow from the *seemingly trivial ulceration* of the os uteri, must certainly be allowed to warrant those who drew it, if only it be a faithful portraiture, in attaching great importance to this affection,—in trying to discover it as early, to cure it as speedily as possible." Here is a part of the "picture":—"It is then inflammation, with its attendant ulceration of the os and cervix uteri, and usually with consecutive induration of its tissue, to which, according to these views, the sufferings of the patients are due; and all the varied disorders of the uterine functions, the pain, the leucorrhœa, the hæmorrhages, the irregular menstruation, the sterility, or the frequently occurring abortions, are attributed to the sympathies of contiguous parts with that small portion of the womb which is the seat of disease." Who has attributed *all* the varied disorders of the uterine functions to inflammation and ulceration of the cervix? Not Dr. BENNET certainly, who no doubt sat for the picture, for neither he nor any one else has alleged more than that this condition of the cervix is a frequent cause of such disorders. Dr. WEST's sneer at the neck as "a small portion of the womb," can be regarded only as a small device to weaken the fortress he wishes to demolish. It is another of his tricks, which I shall presently expose, in endeavoring to assign to the cervix its proper rank in the economy of the sexual organs.

The evidence by which to try the truth of the doctrine, which he has arraigned, is, according to Dr. WEST, very various in its kind and also of very various worth. He arranges it, however, under four principal heads, which I give in full and verbatim, in order that his plan of operations may be fairly unfolded.

"In the *first place* we may enquire how far these statements receive confirmation from what we know of the anatomy and physiology of the uterus in a state of health.

"Still, what answer soever we may receive to this question, it cannot, from its very nature, be conclusive; it may render a certain occurrence probable or improbable, may substantiate or disprove the correctness of certain opinions or explanations, but cannot invalidate the evidence of positive facts.

"In the *second place*, we may try to ascertain whether examination of the dead body shows the morbid conditions of the os uteri which have been described to be frequent or rare, slight or extensive; and we may also endeavor to make out what connection subsists between ulceration of the mucous membrane of the os and cervix uteri, and other changes in the tissue of the organ.

"It must, however, be borne in mind that many evidences of disease, such as are very obvious during life, may be greatly obscured, or may even entirely disappear after death: and further, that uterine disorders of the class which we are considering, though exceedingly painful, and seriously interfering with a woman's health and comfort, are yet not of a kind to prove the direct occasion of her death. Evidence derived from this source will therefore be open to the objection that it understates both the frequency and the importance of these diseases.

"We may enquire, in the *third place*, whether there is any condition in whsch ulceration of the os uteri comes under our notice unconnected with other disease, and with such circumstances as to admit readily of our observing its characters and watching its course and consequences. Such a state of things presents itself to us often in the case of the procident uterus, siuce the irritation to which the displaced organ is unavoidably exposed has the almost invariable effect of producing ulceration of the surface of the os uteri, and of the immediately adjacent parts of the organ.

"But, whatever conclusions we may deduce from this source are open to all the objections inseparable from analogical reasoning. The probabilities of certain occurrences taking place in the uterus under other circumstances may be increased or weakened; but the evidence still falls short of absolute proof, either of the affirmative or of the negative of the question.

"The *fourth* and most important enquiry of all concerns the frequency of these ulcerations of the os uteri under those circumstances in which they ordinarily come under our notice, and call, or are supposed to call, for our interference. This enquiry, however, must include not only the frequency of ulceration, but also the conditions generally associated with it, and the symptoms to which it commonly gives rise. If the alleged symptoms of ulceration are found to be not rarely present without ulceration, and if ulceration is discovered even where there are no symptoms; or if, in the same case, the ulceration may vary in extent, with no corresponding change in the symptoms; if an indurated state of the cervix exists without, ulceration and ulceration even of long standing, without induration,—the conclusion, especially if supported by the answers obtained to our previous enquiries, seems to me irresistible that the importance of inflammation of the cervix and of ulceration of the os uteri has been overstated; that they are not the cause of all the symptoms which they have been alleged to occasion, and that, in the treatment of uterine disease, many other considerations must influence us more than the mere removal of ulceration of the orifice of the womb."

Such is the ground which Dr. WEST proposes to travel over and the different lines of argument he intends to pursue in his attack upon the cervical doctrine, and it will be my task to follow in his footsteps and report, as impartially as I can, the progress of his arms.

His first argument is drawn from what anatomy and physiology teach us of the uterus and its functions, and is a regular attempt to exalt the body and degrade the neck of the organ. He had before, as we have seen, tried to ignore inflammation or merge it in trifling abrasion, and now he thrusts at the seat itself of this unimportant lesion, with the design to throw it entirely into the shade. With this view, he brings out in full relief, the richer vascularity and higher vitality of the body of the womb, compared with the neck. It is from the congested lining membrane of the body of the womb that the menstrual flux is poured out; it is the body of the womb, which first and chiefly enlarges during pregnancy; it is the mucous membrane which is metamorphosed into the decidua and which is detached and reproduced again and again after delivery. And while the corpus uteri is the theater of these wondrous operations, the cervix is comparatively passive; it has no part in menstruation; its follicles secrete a little mucus during pregnancy, and when delivery is over, its stretchod mucous membrane resumes once more its former plicated arrangement. Dr. WEST concludes the parallel by declaring that "the cervix is less sensitive than the body of the uterus; the sound which passes along the canal of the former almost unfelt, generally finds the lining of the uterine cavity acutely sensitive. The cervical canal has been forcibly dilated, it has been incised; the tissue of the cervix has been burnt with the strongest caustics, or with the actual cautery, or portions of it have been removed by the knife, generally with no injurious consequence; often with so slight a degree of constitutional disturbance, or even of local suffering, as to surprise those who advocate, little less than those who condemn such proceedings."

Having thus sunk the cervix to zero in the anatomical and physiological scale, Dr. West has no difficulty in drawing the proper conclusion, which is as follows:—"It certainly does appear to me that the assumption that some slight abrasion of the mucous membrane covering this part is capable of causing a list of ills so formidable as are attributed to it, ought to rest for its support upon some other and stronger foundation than any inference fairly deducible from anatomical or physiological data."

It is essential to a correct understanding of the matters in controversy and, indeed, to right views of uterine physiology and pathology in general, that the cervix uteri be rescued from the degradation into which Dr. West has sought to plunge it. In order to this, it is necessary to consider, *first*, its structure and dimensions, compared with those of the body; *secondly*,, its offices, as a co-operator with the body, in the special functions of menstruation and parturition; and *thirdly*, the kind and degree of sensibility with which it is endowed and its sympathetic influences compared with those of the body.

First. *The structure and dimensions of the cervix.* All the essential anatomical elements that enter into the composition of the body are found likewise in the cervix. It is invested by a peritoneal covering excepting its anterior face, where it is brought into closer companionship with the bladder by reason of its nudity. This closer proximity and the cellular liens that tie it to its urinous neighbor, give to it the power of annoyance and of compelling sympathy with it in its maladies. Hence, the frequent and sometimes painful micturition, almost invariably complained of in cervical inflammation; symptoms, which not unfrequently decoy the physician from the right path of investigation in female ailments. The cervix has a muscular coat like that of the body, which differs only in the greater compactness of its fibers, giving it greater density of structure. These fibers, upon the internal surface of the neck, have a peculiar arrangement, denominated the *arbor vitæ* and contrast strikingly with the internal surface of the body, and this peculiarity, whatever may be its advantages serves to shelter inflammation when it lurks among its branches.

Again: So much of the cervix as projects into the vagina, viz: the os uteri, is covered by mucous membrane, reflected upon it from the vagina and continued, thorugh its external orifice, into its canal,—the whole length of which it lines to the internal orifice. The investing portion of this membrane is as vascular as that of the vagina and not inferior, in this respect, to the mucous membrane of the body, while the lining portion of it is not devoid of blood vessels, though it be not quite so vascular. Throughout the whole of its extent, this mucous membrane is bountifully supplied with follicles, which are especially numerous and large in the cervical canal. Besides these anatomical elements, which it possesses in common with the body, the sub-vaginal portion of the cervix, (the os uteri) according to a late French writer, Dr. FORGET,* receives a layer of *erectile tissue*, which is reflected upon its external surface from the vagina, being a continuation of the erectile coat of the vagina, in the same manner as the mucous membrane of the os uteri is a continuation of the mucous coat of the vagina. In this respect, the os uteri resembles the glans penis, which is constituted, in larger part, of the spongy portion of the urethra expanded upon it. The muscular element of the uterine neck is immediately subjacent to this cortical envelope, as it is called by Dr. FORGET. This analogy of structure in two organs, between which there is such great intimacy of functional relations, is a very interesting fact, should it be confirmed by other anatomists. It affords a more satisfactory explanation, as the same writer has said, of an occurrence sometimes met with in obstetric practice, than the generally received account of the anatomy of the os uteri. The occurrence referred to is an œdematous tumefaction of the free edge of the uterine orifice, at the time of its greatest dilatation during labor, which every one, much employed in this branch of practice, must have observed, and a very remarkable instance of which I have lately

* Etude Pratique et Philosophique du col de la Matrice, Paris, 1849.

met with. Such a swelling, occurring suddenly and disappearing promptly after delivery, may be better comprehended, supposing its seat to be areolar and erectile spongy tissue, than it can be, if the interstices of the muscular fibers, distended and flattened to the utmost degree, are supposed to be occupied by it.

Dr. West's sneering allusion to the cervix, on account of its diminutiveness, has been already noticed, and from it the uninformed might suppose that the neck really is but a very small portion of the uterus. To see how this matter stands, let us refer to the admirable and standard work of Madam Boivin and Duges* for the dimensions of the entire organ and those of its parts, body and neck. They give, as the total length of a virgin adult uterus, accurately measured, 26 lines, of which *exactly one-half* belonged to the neck; width of the fundus, 17 lines; thickness of the fundus, 8½ lines; width of the neck, 9½ lines; thickness of the neck, 7 lines;—from which it will be observed, that the neck is equal to the body in length, and is nearly equal in thickness, but is, of course, narrower across, else it could not be a neck. As to the thickness of their parietes, there is but little difference between the body and neck. Childbearing produces notable changes in the condition of the womb, among which not the least remarkable is the predominance the body acquires over the neck: still the neck is not reduced to insignificant dimensions, for the same authors inform us that while the length of the body is 2 inches, that of the neck is 13 to 15 lines, the other dimensions of the two portions being about equally increased, so that the body's relative gain is only in length.

Secondly. We have to consider the special office of the cervix relatively to menstruation and parturition. In both of these functions, the product to be eliminated is prepared in the cavity of the body, and extruded from it by the contraction of its parietes, the cervical canal being its excretory duct. The healthful performance of both these functions depends, therefore, more than is generally imagined, upon the state of the cervix. If its canal be contracted in any part of its course, whether from malformation or disease, the uterine excretion is rendered difficult, and more pow-

*Traité Pratique des Maladies de l'Uterus et de ses Annexes.

erful expulsive efforts, on the part of the body, are necessitated. To be convinced that this is no exaggerated representation, let us consider these functions for a moment or two. In regard to menstruation, it is now well ascertained by actual observation that the sanguineous part of the discharge exudes from the internal surface of the cavity of the body, and issues slowly through the external orifice into the vagina, where it mingles with the vaginal mucus. Even in the most perfectly healthy condition of the organ, it may be presumed that the fluid is expelled by the contractions of the parietes of the body. Such expulsive efforts, however, are so gentle as to excite no pain and attract no notice. Be this as it may, we have satisfactory evidence that, in morbid states of the function, in dysmenorrhea for example,—obstruction of the cervical canal, may, by rendering the excretion difficult, excite violent, painful, and even spasmodic contractions of the uterine body. It is not to be denied that dysmenorrhea may be attributed to inflammatory action in the body, particularly when membranous and organized substances are discharged; but it is not less questionable that it may be produced by such a purely mechanical cause as the small size of the os uteri, congenital or acquired, contraction of its internal orifice, or diminished caliber of the canal, from inflammation of the substance of the cervix. The interesting cases related by Dr. MACKINTOSH,† which were treated successfully by dilatation with metallic bougies, of different sizes, establish this fact beyond all reasonable doubt. I refer you to his chapter on Dysmenorrhea for the details of these cases, and others might be cited were it necessary. These cervical obstructions, when of long continuance, by exciting such preternatural contractions of the body, at each returning menstrual period, may cause hypertrophy of its walls and inflammation of its lining membrane, in the same way as stricture of the urethra gives rise to these pathological lesions in the bladder.

Again. Spasmodic contraction of the fibers of the cervical canal, during a healthy menstrual flux, may suddenly arrest the flow and cause the menstrual fluid to be retained in the cavity of the body until the next period or for a still longer time. This is

† Principles of Pathology and Practice of Medicine.

one of the obstacles to menstruation, resulting in retention, recognized by Dr. BERNUTZ, in a series of highly interesting articles, entitled "*Memoire sur les Accidents produits par la Retention du Flux Menstruel.*"* The minute recital of several cases, which he has given under this head, leaves no room to doubt that when menstruation is suddenly suppressed during the flow, by exposure to cold or any other cause, it is not always because there is an arrest of the sanguineous exhalation, for this may continue, but because spasm of the excretory canal—the cervix—precludes its escape and compels it to accumulate in the cavity of the body. Some of the facts of these cases, which admit of no other interpretation, may be mentioned here. The sudden stoppage of the discharge is immediately followed by violent pain in the region of the uterus, succeeded by swelling and tenderness. The discharge may not make its appearance at the next period when it is due, but several periods may elapse, and when it is restored, it is accompanied by violent pains, resembling those of labor. The discharge is also more copious, amounting sometimes to hemorrhage, consisting in part of coagula, the first that escape being of a dark color like blood that has been long extravasated.

The important part played by the cervix in the function of parturition is more familiar to the profession, because it obtrudes itself upon our notice in a more palpable manner and presents unmistakable credentials of its active participation. In every instance of childbirth, it opposes some resistance to the contractions of the body, while in some cases, fortunately rare, it is so rigid and unyielding as greatly to protract and exacerbate the sufferings of parturition. Nor must we omit to mention here that the os uteri is not unfrequently lacerated, to a slight extent, in giving passage to the child, and these lacerations, together with the contusion to which it is subjected, often lay the foundation for inflammatory mischief. All who are practically conversant with female diseases, know that many cases of inflammation of the cervix can be traced back to the date of the patient's accouchment, —the characteristic symptoms of the affection being complained of when she first attempted to resume her ordinary occupation.

* Archives Générales de Médecine, 4th series, tome xvii, xviii, xix.

In the third place, in order to do justice to the uterine neck, we are to inquire into its relative sensibility and its sympathetic reaction upon other organs.

All anatomical authors agree that the uterus is abundantly supplied with nerves, derived from the great sympathetic, through the hypogastric plexuses, and that, intermingled with these, are branches of the sacral nerves, arising from the spinal cord. It is, therefore, endowed with ganglionic and cerebro-spinal nerves, or, as they are often designated, nerves of organic and animal life. Most writers, whose authority on such subjects stands deservedly high, such as TIEDEMAN, LEE, MOREAU, VELPEAU, &c., represent that both portions of the organ, body and cervix, are alike supplied with these two orders of nerves, while, in reference to the cervix, M. JOBERT maintains that its superior portion, viz: that which is above the vaginal attachment, receives only organic nerves, and that the subvaginal portion (os uteri) is completely destitute of nervous filaments of any kind, and is absolutely insensible! Between M. VELPEAU, who declares that cerebro-spinal nerves are detached from the hypgastric plexures to be distributed to the neck of the uterus, especially to the os, and M. JOBERT, I cannot pretend to decide on anatomical grounds, for I have never attempted to unravel this knotty point by a minute dissection. I know enough of the anatomy of the uterus, however, to be satisfied that it must be exceedingly difficult to distinguish between numerous delicate filaments, coming from a common plexus, and determine which are organic and which animal, or to trace them, with the scalpel, in their tortuous distribution through the tangle of closely complicated fibers, composing the muscular coat of the organ. I have but little faith in the dissection of any anatomist on such a point, and none at all when its results are contradictory of clinical observation. What evidence more conclusive of the possession of nerves can be given by any organ or tissue than the exhibition of sensibility under the application of tests calculated to elicit it? Tried by this method of proof, what answer does the os uteri return to our inquiries concerning its sensibility? Can it be cut or excised, lacerated or burnt, without feeling, and without exciting any sympathetic reaction in other organs of the system? Let facts reply to this question. The celebrated French surgeon, Lisfranc, amputated the vaginal portion of the neck, in a large number of cases of uterine disease, and we are informed by one of his pupils, Dr. Forget, that he was accustomed, in the midst of the operation, to interrogate his patients as to the nature of the sensation they experienced. From such inquiries he learned that the pain felt by a majority of them was not acute, but they all complained of *some unusual and disagreeable sensation:* with some, it was that of a very hot iron passing over the depth of the sexual parts; with others, it was a sort of setting on edge, (*agacement*) difficult to express but profoundly felt; with a certain number, it was a genuine uterine colic, of moderate intensity. The conclusion to which the surgeon of *la Pitié* came, as the result of his numerous inquiries, was, that if the os uteri showed but little sensibility to cutting, it is much more sensitive to pressure, resembling the testicle in respect to its mode of sensibility.

With regard to the application of caustics to the os uteri, my own experience warrants me in speaking positively, and I do not hesitate to affirm that, whether the nitrate of silver, the acid nitrate of mercury, or the potassa cum calce be used, more or less pain is immediately felt, and continues to be complained of during the succeeding twenty-four hours. The pain is sometimes exceedingly violent, in the form of uterine spasms, requiring large doses of laudanum or morphia, with brandy and hot applications, for its subdual. It is worthy of remark that the pain, excited by topical applications to the os uteri, is often only an exacerbation of the sacral, iliac, or hypogastric pain, habitually complained of by the patient. I have assured myself of the truth of this assertion, by such repeated and particular inquiries, that there can be no mistake as to the identity of the pains of inflammation and those of cauterization of the os uteri, which may, I think, be regarded as little less than a demonstration of the precise local source of these pains.

Careful inquiries have satisfied me that the os uteri is sensible to pressure. In examinations by the touch, when the finger is pressed upon its apex, it is scarcely felt by the patient, or it may not be felt at all; but when pressure is made upon either side of the os, as high up as the vaginal attachment, it is always felt, and if there be inflammation, it is painfully felt, which may be regarded as one of the diagnostic marks of this morbid condition.

Finally. Were there no other evidence of the sensibility of the cervix, carcinoma, limited strictly to this portion of the uterus, affords convincing proof, in the cruel pain which attends its distructive progress, sufficient to put to flight the doubts of a host of anatomists.

It is an opinion, very generally entertained, that the cavity of the body is much more sensitive than that of the neck. It is very positively affirmed by Dr. WEST; and yet it does not at all accord with my own experience. I have often introduced the sound into the uterine cavity to its fundus and have carefully noticed the expression of the patient during the operation. The passage of the sound is not free from pain, either while it is traversing the cavity of the neck or that of the body; the pain is not, however, very acute along the tract of either cavity, and I have not been able to perceive that there is any difference in its degree. Moreover, I have often cauterized both cavities with the nitrate of silver, and sometimes, though rarely and very cautiously, with the potassa cum calce, and have not discovered, by this test, any difference in their sensibility.

If, then, the os and cervix possess the same nervous endowment as the body, there can be no reason to doubt that they share with it the influence which the uterus is known to exert over other organs that sympathize with it; and in proof of this declaration, I may refer to one of the uterine sympathies, which I have, more than once, known to be awakened by irritation of the cervix. In some patients, effected with chronic phlegmasia of the neck, nausea is among the most prominent symptoms complained of, which they declare is like the nausea of early pregnancy. In such cases, firm pressure with the finger upon the cervix or the application of caustic may immediately excite nausea, just as the same treatment revives and aggravates the sympathetic pains attendant upon cervical inflammation.

The statements and facts, which have now been produced, are sufficient, I think, to blunt the edge of the argument from anatomy and physiology, as urged by Dr. West against the importance of the cervix in uterine pathology. Let us now try whether or not his second argument consists of better metal. The argument, it will be remembered, is deduced from morbid anatomy, being the result of *post mortem* investigations to ascertain whether pathological conditions of the os uteri are frequent or rare, slight or extensive. In the outset of his remarks on this branch of the subject, Dr. West observes: "It seems somewhat strange that those who believe in the frequency and importance of ulceration of the os uteri, have made no attempt to demonstrate those facts by examination of the body after death; while the only persons who have appealed to its results, allege this condition to be very rare and very trivial." This is truly a curious bewilderment of our Croonian lecturer. To me it appears the most natural thing in the world, that those who have abundance of *living* evidence should refrain from seeking corroboration of it among the dead. Why does it not seem equally strange to Dr. West that no *post mortem* evidence is sought for or deemed necessary in cutaneous inflammation or ulceration? The cases are precisely on a par; it is as easy to learn by the speculum whether or not there is inflammation or ulceration of the os uteri, as it is by direct inspection to satisfy ourselves whether or not the skin is the seat of these morbid conditions. In either case, all examination of the dead body to determine the simple question of the existence of ulceration would be not only supererogatory, but ridiculous. But notwithstanding my inability to comprehend the necessity or utility of this branch of his investigations, I am not loth to accompany Dr. West and listen to the voices of the dead, in response to his gratuitous interrogatories.

Previous researches of this kind appeared to authorize the conclusion that ulceratiou of the os uteri is of very rare occurrence,—M. Lair, for example, quoted by Dr. West, having met with it but 12 times in 500 examinations, and M. Pichard 5 times in 300. Dr. West, however, objects to these examinations, and also those made in England, that they were carelessly made, and without the least information as to when, where and how, or as to the age of any of the subjects. The latter omission he regarded as a capital defect, and charges that in his own country there has been such total want of discrimination that "conclusions have been drawn from the examination alike of the infant of a few weeks old and of the old woman of seventy." If his countrymen have pressed babies into service, in these investigations, they have, indeed, been less discriminating than they usually are; but I do not clearly perceive the propriety of selecting subjects exclusively from females during the child-bearing period of life, as Dr. WEST has done, for although it may be true that the period of greatest functional activity is also that of the greatest liability to diseases of the sexual organs, it cannot be pretended that these organs are absolutely exempt, at an earlier or later period, from such pathological lesions as are common to all others.

Dr. WEST's observations amount to only 62, but he hopes that the care with which they were made will compensate to some extent for the smallness of their number, and the uteri were taken from patients who died in the medical wards of St. Bartholomew's Hospital of other than uterine disease. "Of the total number," he informs us, "13 were above forty-five years of age, the remaining 49 between the years of fifteen and forty-five. Concerning all of the former class and 30 of the latter, making a total of 43, it was either known with certainty, or concluded with great probability, that they were married or had had sexual intercourse; the remaining 19 were believed to be virgins."

The general results of the examination of the uterus in these cases are given by Dr. WEST in tabular form, which is subjoined.

TABLE,

*Showing the chief results of the examination of* 62 *uteri:—*

| | | |
|---|---|---|
| Uterus healthy in - - - - - - - - | | 33 |
| " diseased in - - - - - - - - - | | 29 |
| Ulceration of os uteri in - - - - - - - | | 17 |
| " existed alone in - - - - - - - | 11 | |
| " with diseased lining of uterus in - - - | 3 | |
| " with induration of walls of uterus in - - | 3— | 17 |
| Induration of walls of uterus, without ulceration of os - - | | 5 |
| Disease of lining of uterus, without ulceration of os - - | | 7 |
| Total of diseased uteri, | | 29 |

In commenting on this table, Dr. West observes that there is something "at first" not a little startling in its disclosures, showing, as it does, that the womb was found in a diseased condition in nearly one-half of these women, none of whom died of uterine disease. But he is startled only for a moment, for it soon occurs to him that some of these morbid appearances may be of little moment, "and," says he, putting on his philosophical glasses, "the very frequency of their occurrence, instead of substantiating the opinion that they are of great importance, rather militates against that supposition. When ulceration of the os uteri was first observed, it was natural enough to attribute to it many symptoms, and to refer to its influence many structural changes. But what if such ulceration be found to be usually very limited in extent, and *so superficial* as to be unassociated with changes in the basement membrane of the affected surface, and exercising so little influence on the state of the uterus in general, as to be unconnected in a large number of instances with changes either in the interior of the womb, or in its substance; while induration of the uterine tissue and disease of the lining membrane of the womb are found independent of it, or of each other? Should such appear to be the case, it will, I think, be rendered in the highest degree probable that *this abrasion* of the os uteri has not the long train of sequences which have been supposed to follow it, but that it is of comparatively small pathological import; that it may be found to vary under the influence of comparatively trifling causes; and not unfrequently to be *dependent* on functional disorder of the uterus, just as the mucous membrane of the tongue and mouth betrays the disturbance of the digestive system; that it may, in short, be the consequence, and sometimes the index, but rarely the occasion, of the ailments with which it is associated."

Dr. West concludes his first lecture with a more minute description of the morbid or pseudo-morbid appearances of the uterus, in these cases, and an attempt to determine their relations to one another, especially that of induration to ulceration, between which, he thinks, there exists no necessary connection, as the former may exist without the latter. Instead of engaging at present in a controversy with him on this point, the determination of which is of no particular consequence, and is, perhaps, impossible, at present, for want of sufficient data, I prefer to adhere to the matter more immediateiy in hand, namely: to inquire what light morbid anatomy has shed on the naked question of the frequency, merely, of ulceration of the os uteri.

To assist in solving this question, I shall be under the necessity of calling upon Dr. ROBERT LEE to depose, and if his testimony shall be found contradictory to that of Dr. WEST, there is no fear of hostile collision, for they are, doubtless, good friends, and have joined heart and hand to accomplish the same object, viz: *to abolish ulceration of the os uteri, and with it the speculum uteri.* The tendency of Dr. WEST's performance is obvious enough, but he is careful to display no hostility to the speculum, any where in his lectures; the conclusion to which the reader, drifted in his current, is, however, inevitably conducted, is, that if ulceration be such a trivial affection, and plenty, withal, as blackberries, there is no need of the speculum to detect or cure it.

Dr. LEE is not so wily an adversary: he openly avows his aims and denounces, in no measured terms, the speculum, in most of the uses to which it has been applied.

*Dr. Robert Lee on the witness-stand.* At a meeting of the Royal Medical and Chirurgical Society, Tuesday, May 28th, 1850,—the rooms being unusually crowded with fellows and visitors, a paper was read by Dr. ROBERT LEE, on the use of the speculum, in which *post mortem* statistics, quite imposing by their grandeur, are brought forward to prove that there is hardly such a disease as simple ulceration of the os uteri, which, we are informed, accords also with Dr. LEE's experience in the living, he having met with a considerable number of cases in which it had been affirmed by others to exist, after deliberate and repeated examination by them with the speculum, where he ascertained that ulceration did not exist in the os and cervix uteri, nor disease of any kind. But to the autopsic testimony:

"In the year 1832," he says, "my colleagues at the St. Marylebone Infirmary,—Dr. Hone, Dr. Sims, Mr. Stafford, and Mr. Perry, late secretary of the Society,—at my request, desired that the uteri of all the women who died in the wards should be carefully examined, and that they should be preserved for my inspection when any morbid appearance was observed. From 1017

post-mortem examinations of females of all ages, made by Dr. Boyd, (after deducting those of children and others, in which special mention is not made of the uterus.) there were found 708 where either the state or weight of the uterus was noted. In thirteen of these there was congestion or inflammation, which had no specific character, and in some the inflammation was limited to the fundus, and could not have been detected unless the uterus had been removed or cut open. In at least three there were enlargement and induration, which did not appear to have any specific character, and in two there was extreme wasting; twenty-four were puerperal cases, thirteen dropsy of the ovaries or Fallopian tubes, in thirty-one fibrous or bony tumors, and in twenty-one, cancer." "My impression is," adds Dr. Boyd, in the same report, "that ulceration of the neck or mouth of the womb is an exceedingly rare disease, else I must," he says, "have observed it, having cut up and weighed many hundreds; it could scarcely have escaped my notice."

"Dr. Allen, the present resident medical officer at the St. Marylebone Infirmary, has held the office about twelve years, and he states to me that he has examined, or been present at, the examination of the bodies of more than 1000 adult females, and of these he does not believe that he ever saw more than twenty examples of ulceration of the os uteri of any kind, scrofulous or venereal, excluding cases of ulcerated cancer of the uterus, which were known to exist before death. Dr. Allen further states, that he has observed in some cases a portion of the mucous membrane of one lip slightly abraded; this he has seen occasionally, but not often. Mr. Prescott Hewett was six years curator of the Museum of St. George's Hospital, and conducted all the post-mortem examinations. He states, that during that time he could not have examined fewer than 600 uteri, and very seldom, if ever, did he meet with anything which could be called ulceration of the os and cervix uteri, independently of scrofula and cancer. Mr. George Pollock held the same office for three years, during which time he examined the bodies of 300 women, and in every case the uterus was cut open and examined. In four cases uterine ulceration was observed, but three of these were scrofulous patients, and scrofulous ulceration existed in other organs. In the fourth case the ulceration must have been cancerous, as it involved the vagina extensively, as well as the os uteri. Mr. Hewett and Mr. Pollock did not, therefore, observe a single example of simple ulceration of the os and cervix uteri in the 900 uteri they examined, which confirms the accuracy of the opinion given by Dr. Boyd, that ulceration of the neck or mouth of the womb is a very rare disease. Mr. Gray succeeded Mr. Pollock at St. George's Hospital, and he examined 180 uteri. Distinct ulceration of the os and cervix was only observed by him in three uteri, and the nature of the ulceration in these three cases was not determined with certainty. Mr. Gray states to me, further, that redness, slight abrasions, and granulations, were sometimes, but not frequently, observed."

From this extract, it will be perceived that none but the bodies of adult females were examined in reference to the point in controversy, and notwithstanding that there must have been among them many belonging to the fruitful period of life, inflammation or ulceration of the os uteri was found in but a small proportion of them, proving the remarkable immunity of the os uteri from such affections. How are we to reconcile this discrepancy between Dr. LEE and Dr. WEST? How, but by supposing that each found what he sought, and what he judged would be most effective in arresting the progress of the speculum. Prejudice is proverbially blinding in its influence, and under its dominion Dr. LEE and those who saw for him may not have seen what was plainly before their eyes; but it may likewise sharpen the sight, as is the case of Dr. WEST, and enable it to see what is hid from others. We may, however, without perplexing ourselves for a rationale, leave the difficulty to be adjusted between them, content with drawing an inference, which is irresistible, viz: that *post-mortem* evidence, thus far, is neutralized by its contradictions, and therefore, even were it admissible in this controversy, it must, in all fairness, be rejected.

